# Establishment and validation of a clinical model for predicting diabetic ketosis in patients with type 2 diabetes mellitus

**DOI:** 10.3389/fendo.2022.967929

**Published:** 2022-10-19

**Authors:** Mengmeng Qi, Xianfeng Shao, Ding Li, Yue Zhou, Lili Yang, Jingwei Chi, Kui Che, Yangang Wang, Min Xiao, Yanyun Zhao, Zili Kong, Wenshan Lv

**Affiliations:** ^1^ Department of Endocrinology and Metabolism, The Affiliated Hospital of Qingdao University, Qingdao, China; ^2^ School of Public Health, Qingdao University, Qingdao, China

**Keywords:** risk prediction model, type 2 diabetes, diabetic ketosis, cross-sectional study, endocrinology

## Abstract

**Background:**

Diabetic ketosis (DK) is one of the leading causes of hospitalization among patients with diabetes. Failure to recognize DK symptoms may lead to complications, such as diabetic ketoacidosis, severe neurological morbidity, and death.

**Purpose:**

This study aimed to develop and validate a model to predict DK in patients with type 2 diabetes mellitus (T2DM) based on both clinical and biochemical characteristics.

**Methods:**

A cross-sectional study was conducted by evaluating the records of 3,126 patients with T2DM, with or without DK, at The Affiliated Hospital of Qingdao University from January 2015 to May 2022. The patients were divided randomly into the model development (70%) or validation (30%) cohorts. A risk prediction model was constructed using a stepwise logistic regression analysis to assess the risk of DK in the model development cohort. This model was then validated using a second cohort of patients.

**Results:**

The stepwise logistic regression analysis showed that the independent risk factors for DK in patients with T2DM were the 2-h postprandial C-peptide (2hCP) level, age, free fatty acids (FFA), and HbA1c. Based on these factors, we constructed a risk prediction model. The final risk prediction model was L= (0.472*a* - 0.202*b* - 0.078*c* + 0.005d – 4.299), where *a* = HbA1c level, *b* = 2hCP, *c* = age, and *d* = FFA. The area under the curve (AUC) was 0.917 (95% confidence interval [CI], 0.899–0.934; *p*<0.001). The discriminatory ability of the model was equivalent in the validation cohort (AUC, 0.922; 95% CI, 0.898–0.946; *p*<0.001).

**Conclusion:**

This study identified independent risk factors for DK in patients with T2DM and constructed a prediction model based on these factors. The present findings provide an easy-to-use, easily interpretable, and accessible clinical tool for predicting DK in patients with T2DM.

## Introduction

Diabetes mellitus (DM) and its related complications are regarded as a major global health threat. The International Diabetes Federation predicts that the global diabetes prevalence in individuals aged 20–79 years will rise to 12.2% (783.2 million) by 2045 (1). Type 2 DM (T2DM) accounts for over 90% of DM cases (2). Diabetic ketosis (DK) is an acute complication of T2DM, but is preventable (3). However, despite the development of new therapeutic drugs and improvements in diabetic care, DK remains one of the leading causes of hospitalization among patients with diabetes (4, 5). Failure to recognize the symptoms of DK can lead to complications, including diabetic ketoacidosis (DKA), severe neurological morbidity, and death (6, 7). A recent study analyzed the National Inpatient Sample database for all DKA admissions in the USA between 2003 and 2014 and found a significant increase (56%) in the inflation-adjusted hospital charges for DKA admissions during that timeframe (4). Early diagnosis and effective management of DK are essential for delaying disease progression, reducing its economic impact, and lowering the risk of complications (8).

DK can develop in patients with diabetes who experience an increase in the level of ketone bodies in the blood, which is a consequence of both the increased ketone production in the liver and reduced urinary clearance of ketones (9). There is growing evidence that a ketogenic diet is therapeutically beneficial for neurologic diseases (10, 11). However, when the concentration of ketones becomes too high, they interfere with normal cellular function. Consequently, hyperketonemia with or without acidosis is an acute severe complication of poorly controlled or newly diagnosed DM. Thus, ketones play an important pathophysiological role in the development of both diabetes and diabetic complications (12).

While the importance of early detection and prevention of DK is known, there have been no population-based studies aiming to develop a clinical model for predicting DK in patients with T2DM. A predictive model for a DK diagnosis, based on clinical information, could improve the effectiveness of T2DM management if the risk for DK is known. Thus, we designed a cross-sectional study to assess patients with T2DM in our hospital, with the aim of developing a DK prediction model for patients with T2DM.

## Methods

This retrospective cross-sectional study was approved by the Ethics Committee of The Affiliated Hospital of Qingdao University (Approval No. QYFYWZLL26666). This study was conducted and reported in accordance with the guidelines of the Transparent Reporting of a multivariable prediction model for Individual Prognosis or Diagnosis (TRIPOD) initiative and the Declaration of Helsinki.

### Study design and patient selection

Data were collected from hospitalized patients with T2DM at The Affiliated Hospital of Qingdao University (Shandong, China) from January 1, 2015, to May 1, 2022. Selected study patients (3,061 patients in total) met the American Diabetic Association (ADA) 2014 criteria for T2DM. Of these patients, 309 had ketosis and 2,752 did not. The diagnosis of ketosis was based on having moderate to high levels of urine ketones, in accordance with the ADA 2009 guideline (13). A computer system was used to randomly allocate 70% of the patients into a cohort for model development and the remainder were moved into a validation cohort.

### Outcomes and variables

The primary outcome of the study was the incidence of DK, which was defined as a severe, acute complication of DM. The patients’ medical files provided anthropometric parameters, including age, BMI, DM duration, WC (waist circumference), and sex. The following laboratory values were also available, including the systolic and diastolic blood pressures and levels of low-density lipoprotein cholesterol (LDL-C), total cholesterol (TC), triglycerides (TG), free fatty acids (FFA), glycosylated hemoglobin (HbA1c), alanine aminotransferase (ALT), aspartate aminotransferase (AST), serum creatinine (sCr), serum uric acid (sUA), fasting plasma glucose, fasting plasma insulin, 120-min postprandial insulin (Ins120), 2-h postprandial C-peptide (2hCP), and C-peptide (CP).

### Statistical methods

The baseline characteristics of the development and validation cohorts were analyzed. Normally distributed data are presented as the mean and standard deviation; non-normally distributed data are presented as the median and interquartile range. Comparisons between the DK and non-DK (NDK) groups were performed using parametric or nonparametric tests (i.e., chi-square, Student’s t-test, and Mann–Whitney U test), as appropriate.

The most relevant patient variables from the univariable analysis were used in the final multivariable logistic regression model. The significant variables from the first analysis, as well as those that could be of clinical interest, were included in the multivariate logistic regression analysis. Variables with collinearity or that were a linear combination of other variables were eliminated.

The regression coefficients were used to construct a DK prediction model, in which the dependent variable was the presence or absence of DK. The calibration was assessed using the Hosmer–Lemeshow test, and model discrimination was assessed by analyzing the area under the curve (AUC) of the receiver operating characteristic (ROC) curve for each imputed dataset. The final model was regarded as having optimal clinical significance. Internal validation was used to evaluate the discrimination of the model (14). All statistical analyses were performed using SPSS Version 20.0 (IBM Corp., Armonk, NY, USA).

## Results

### Study population

**Figure 1:**
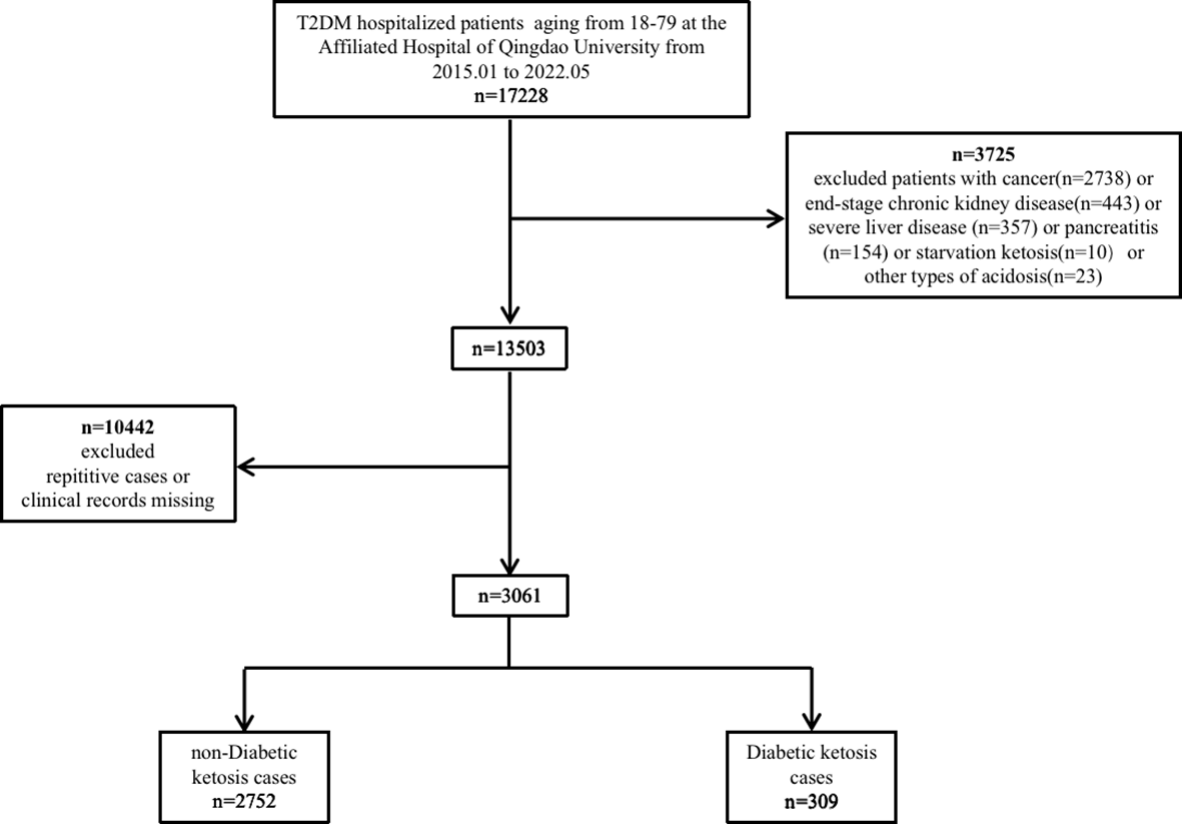
Flowchart of the inclusion and exclusion of participants.

This study included 3,061 hospitalized patients who were treated at The Affiliated Hospital of Qingdao University between January 1, 2015 and May 1, 2022 (**Figure 1**). A computer system randomly assigned 2,156 patients to the development cohort and the remainder (n = 905) to the validation cohort, with 10.1% and 10.1% DK prevalence in each cohort, respectively.

### Prediction model

**Table 1** shows the baseline characteristics for the DK and NDK datasets of patients with T2DM.

**Table 1:** Baseline characteristics of patients with and without ketosis in T2DM.

Factors	NDK (n=2752)	DK (n=309)	P
**Sex**			
** Male**	1499	192	0.010
** Female**	1253	117	
**age, y**	60.0 (53.0,66.0)	45.0 (33.0,57.0)	<0.001
**BMI, kg/m^2^**	26.2 ± 3.85	26.7 ± 4.84	0.049
**WC, cm**	95.3 ± 10.5	94.9 ± 13.1	0.599
**DM duration, y**	10.0 (4.00,15.0)	5.00 (1.00,10.0)	<0.001
**BP, mmHg**			
** SP**	140 ± 18.8	136 ± 18.0	0.001
** DP**	79.6 ± 11.7	81.7 ± 11.5	0.003
**CP, ng/ml**	1.90 (1.20,2.70)	1.20 (0.700,1.70)	<0.001
**CP120, ng/ml**	3.70 (2.30,5.80)	2.00 (1.10,3.40)	<0.001
**Ins, uIU/mL**	7.95 (4.80,13.3)	5.30 (2.70,9.55)	<0.001
**Ins120, uIU/mL**	20.6 (10.4,37.3)	12.9 (5.35,29.2)	<0.001
**HbA1c, %**	8.28 ± 1.90	11.0 ± 2.54	<0.001
**FPG, mmol/L**	7.10 ± 2.34	10.2 ± 4.27	<0.001
**Lipid Profile, mmol/l**			
** HDL-C**	1.20 ± 0.313	1.12 ± 0.316	<0.001
** LDL-C**	2.72 ± 0.946	2.97 ± 0.977	<0.001
** TG**	1.40 (1.00,2.10)	1.60 (1.00,2.70)	0.001
** TC**	4.57 ± 1.21	5.05 ± 1.64	<0.001
**FFA, umol/l**	400(270,490)	581(520,600)	<0.001
**sCr, umol/l**	56.0 (47.0,69.0)	61.6 (48.5,77.5)	0.002
**sUA, umol/l**	333 ± 93.4	322 ± 115	0.099
**ALT, IU/L**	18.0 (13.0,25.0)	19.0 (13.0,28.7)	0.144
**AST, IU/L**	17.0 (14.0,21.0)	16.0 (12.5,23.0)	0.190

Normally distributed variables are expressed as mean ± standard deviation, non-normal variables are expressed as median (IQR) and categorical variables are expressed as percentage (%).

BMI, Body Mass Index; SP, systolic pressure; DP, diastolic pressure; CP, C-peptide; 2hCP, 2hour postprandial C-peptide; HbA1c, hemoglobin A1c; Ins, Insulin; Ins120, Postprandial 120minutes insulin; LDL-C, low-density lipoprotein cholesterol; FFA, free fatty acids; TC, total cholesterol; TG, triglycerides; sCr, serum creatinine; sUA, serum uric acid; ALT, Alanine aminotransferase; AST, Aspartate aminotransferase; WC, waist circumference; DM, diabetes mellitus.

The strongest predictors of DK incidence in patients with T2DM were age, DM duration and CP, 2hCP, HbA1c, FPG, LDL-C, TC, FFA, TG, ALT, and AST levels (**Table 2**). The result of multivariable logistic regression was showed at **Table 3**. Considering their statistical and clinical significance, the four variables selected for the final model were age and the levels of HbA1c, FFA, and 2hCP.

**Table 2:** Univariable logistic regression of patients with and without ketosis in T2DM.

Factors	Univariate analysis		
	Odds ratio	95% CI	P
**age, y**	0.920	0.910-0.932	<0.001
**BMI, kg/m^2^**	1.059	1.025-1.094	0.001
**WC, cm**	0.999	0.986-1.013	0.940
**DM duration, y**	0.919	0.898-0.941	<0.001
**Blood Pressure, mmHg**			
** SP**	0.988	0.980-0.996	0.002
** DP**	1.016	1.004-1.027	0.009
**CP, ng/ml**	0.512	0.433-0.605	<0.001
**CP120, ng/ml**	0.728	0.674-0.786	<0.001
**Ins, uIU/mL**	0.995	0.986-1.003	0.219
**Ins120, uIU/mL**	0.997	0.993-1.001	0.193
**HbA1c, %**	1.734	1.615-1.861	<0.001
**FPG, mmol/L**	1.401	1.334-1.472	<0.001
**Lipid Profile, mmol/l**			
** HDL-C**	0.342	0.207-0.563	0.005
** LDL-C**	1.293	1.130-1.478	<0.001
** TG**	1.108	1.048-1.172	<0.001
** TC**	1.266	1.152-1.392	<0.001
**FFA, umol/l**	1.004	1.003-1.005	<0.001
**sCr, umol/l**	1.001	0.996-1.006	0.607
**sUA, umol/l**	0.999	0.998-1.001	0.278
**ALT, IU/L**	1.013	1.006-1.020	<0.001
**AST, IU/L**	1.020	1.010-1.031	<0.001

The relationship with the DK risk in the univariate logistic regression model is expressed as the odds ratio (OR) and its 95% confidence interval (CI).

**Table 3:** Multivariable logistic regression of patients with and without ketosis in T2DM.

Factor	Multivariate analysis		
	Odds ratio	95% CI	P
**age, y**	0.926	0.911-0.942	<0.001
**CP120, ng/ml**	0.818	0.739-0.905	<0.001
**HbA1c, %**	1.597	1.456-1.751	<0.001
**TG, mmol/l**	0.937	0.862-1.019	0.130
**FFA, umol/l**	1.005	1.004-1.006	<0.001
**DM duration, y**	0.975	0.946-1.005	0.099
**ALT, IU/L**	0.996	0.986-1.006	0.407

The relationship with the DK risk in the multivariate logistic regression model is expressed as the odds ratio (OR) and its 95% confidence interval (CI).

**Figure 2:**
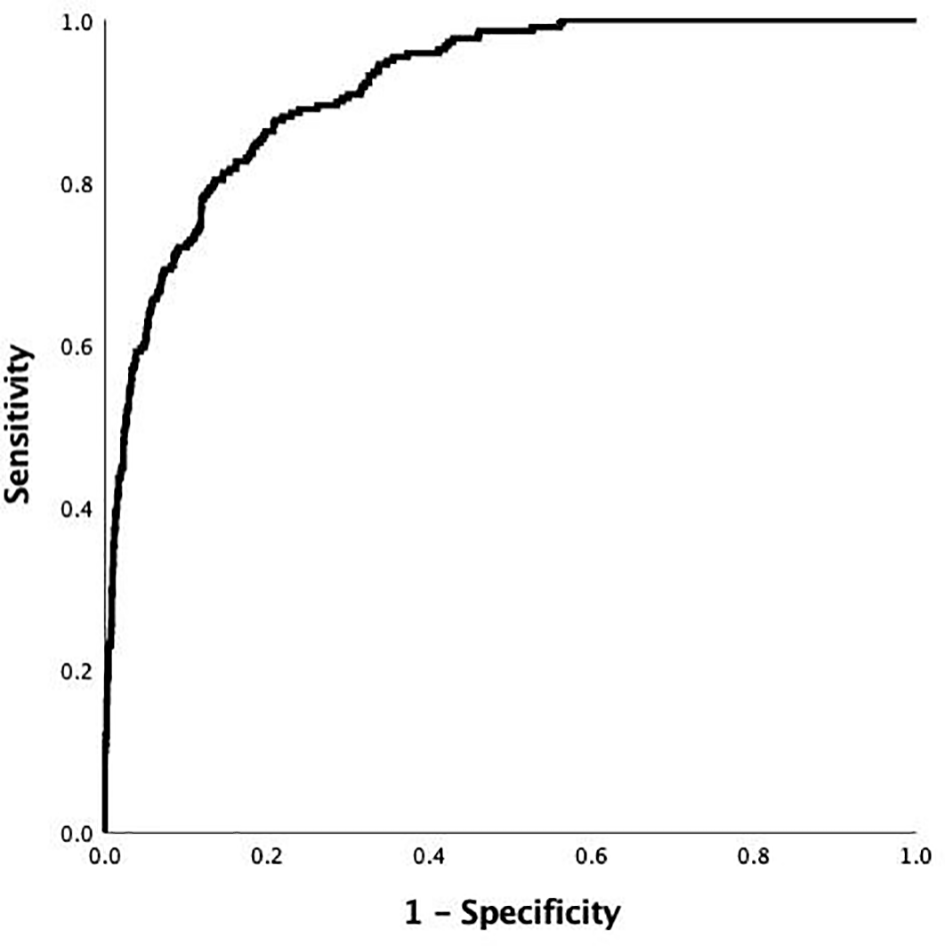
Receiver-operating characteristic (ROC) curve of the risk prediction model of DK in patients with T2DM in the development cohort. The AUC and its 95% CI were 0.917 (0.899-0.934).

The final model was L= (0.472*a* - 0.202*b* - 0.078*c* + 0.005d – 4.299), where *a* = HbA1c level, *b* = 2hCP, *c* = age, and *d* = FFA. The median AUC was 0.917 (95% confidence interval [CI]: 0.899–0.934) (**Figure 2**). The largest Jordan index was 0.667, with a sensitivity of 0.876 and specificity of 0.791.

### Model performance

**Figure 3:**
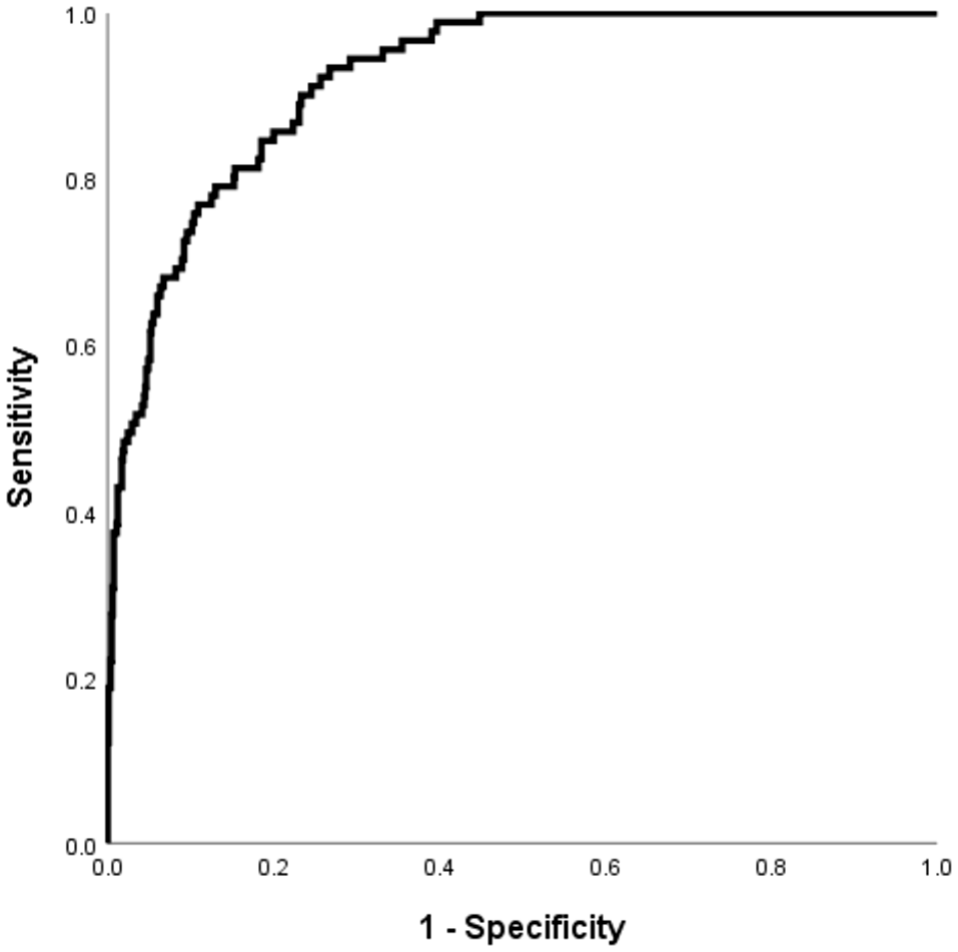
The performance of the DK in patients with T2DM risk prediction model in the validation cohorts. Receiver operating characteristic curve for the DKD risk prediction model. The AUC and its 95% CI were 0.922 (0.898-0.946).

The Hosmer–Lemeshow test, which is a goodness-of-fit test for logistic regression, showed significance for all imputed datasets (p =0.833). The calibration curve for the predicted and observed DK is shown in **Figure 3**.

### Internal validation

**Figure 4:**
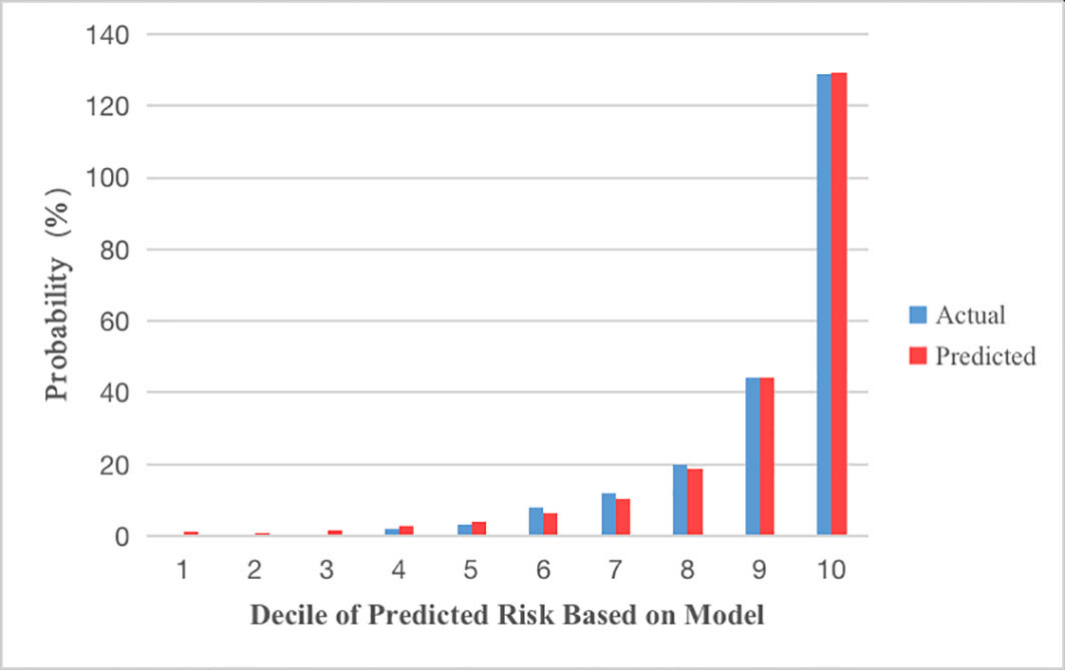
Calibration plots show the relationship between the predicted probabilities base on the prediction model and actual values of the development cohorts. The x-axis represents deciles of predicted risk, and the y-axis reveals predicted and actual prevalence of DK. The H-L chi-square which measure the calibration was 4.255 (P = 0.833).

Using the validation cohort dataset, an ROC curve for the DK risk prediction model was generated and is shown in **Figure 4**. The AUC was 0.922 (95% CI: 0.898–0.946) in this cohort. Given that the aim of developing the model was the early detection of patients with T2DM who are at high risk for DK, a value of -2.779 was selected as the optimal cutoff risk score, which had a sensitivity of 0.901 and a specificity of 0.767.

## Discussion

This study developed the first predictive model for DK in patients with T2DM. The final predictive model included four variables: age and the levels of 2hCP, FFA, and HbA1c. At present, there are several models for predicting T2DM complications such as nephropathy and diabetic retinopathy (15, 16). To the best of our knowledge, this is the first study to suggest a model for predicting the diagnosis of T2DM with ketosis (T2DK).

Early studies identified ketosis as a complication of type 1 DM (17). However, evidence shows that ketosis occurs more frequently in patients with T2DM. Pancreatic islet cell dysfunction is thought to cause ketosis; individuals who are more susceptible to ketosis tend to have poorer pancreatic islet function (18). Changes in CP levels are pathophysiologically typical in patients with DM and are commonly used to evaluate islet β-cell function (19). The interpretation of a CP test can assist in predicting ketosis and inform recommendations for patient treatment options. In addition, studies have confirmed a predominance of postprandial glycemia in the overall glycemic control of patients with well-controlled T2DM who are managed using oral hypoglycemic agents or basal insulin (20). This may explain why 2hCP was one of the variables identified for inclusion in our final model.

In recent years, the importance of HbA1c levels has been increasingly focused on for the diagnosis of diabetes. Hallberg et al. (21) showed that HbA1c levels are an important indicator in the management of diabetes. The level of HbA1c, rather than the fasting glucose level, is a more stable long-term indicator of blood glucose levels over a period of 2–3 months. It also correlates well with the risk of long-term diabetes complications such as acute illness and infection. The HbA1c level is now considered to be the best method for predicting glycemia-associated risks for DM complications (22). Ketosis in T2DM is often caused by poor control of blood glucose levels; based on this, we incorporated the HbA1c level into our model. For decades, FFA has been an important risk factor for insulin resistance, defective insulin secretion, glucose intolerance, and T2DM (23, 24). In our statistical analysis, T2DK patients have increased FFA levels and decreased insulin secretion, which were consistent with previous studies (25, 26).

Several studies have reported higher blood ketone levels in younger patients with T2DM, a finding that is consistent with the results of our study (27, 28). Furthermore, there is accumulating evidence showing that relatively young patients with T2DM have a more aggressive disease phenotype, which often leads to premature development of complications. This can have adverse effects on a patient’s quality of life and unfavorable effects on long-term outcomes (29, 30). In this study, we found that patients in the DK group were younger, on average, than those in the NDK group (p <0.001); this is consistent with previously reported results. We decided to include age in our model, instead of the duration for which patients had diabetes, in consideration of the high rate of undiagnosed diabetes.

Ketosis in patients with T2DM is often overlooked because its symptoms can be atypical (31). However, we found that age and the levels of 2hCP and HbA1c were closely associated with and predictive of ketosis in patients with T2DM. An advantage of our predictive model is that the development and validation datasets are from the same database, which avoids bias and increases predictive efficiency. For the three indicators included in the model, none of the patients had missing values.

In addition, we found that DK is more common in younger patients with diabetes and a higher proportion of male patients have poor pancreatic islet function compared to NDK patients; this is consistent with the results of other studies (28). Previous studies have shown that the ketogenic function is impaired in patients with T2DM, which is related to high serum insulin levels (32). High insulin levels directly inhibit the ketogenic function of the liver; they also inhibit the secretion of growth hormone and glucagon, both of which may contribute to the inhibition of ketone production. Consistent with this information, our logistic regression analysis revealed low insulin as a risk factor for ketosis in patients with T2DM.

Our study had inherent limitations due to the cross-sectional design. It was not possible to identify the cause of ketosis in patients with T2DM. In addition, all patients in this study were enrolled in one center, which may limit its generality. Finally, this study is that some potential risk factors for T2DK may have been overlooked or absent altogether in the patient population. Taken together, a multi-center cohort study to identify the risk factors of T2DK is necessary. It would be helpful to verify the findings of this study using a cohort of patients who have long-term follow-up data with repeated measurements of ketone bodies, and collecting the data in detail would have improved the quality of this study.

## Conclusions

The rapidly increasing prevalence of T2DM has resulted in its identification as an international health concern. T2DM has a major effect on global morbidity and premature mortality, and it is an increasing economic burden due to chronic complications (33). This study found that age and the levels of 2hCP, FFA and HbA1c were correlated significantly with the prediction of T2DK. Moreover, this study developed a satisfactory predictive model with promising application for clinical practice.

## Data availability statement

The raw data supporting the conclusions of this article will be made available by the authors, without undue reservation.

## Ethics statement

The studies involving human participants were reviewed and approved by the research ethics committee of the Affiliated Hospital of Qingdao University (Approval No. QYFYWZLL26666).

## Author contributions

All authors contributed to the study conception and design. Material preparation, data collection and analysis were performed by MQ, XS and DL. The first draft of the manuscript was written by MQ and all authors commented on previous versions of the manuscript. All authors contributed to the article and approved the submitted version.

## Acknowledgments

We thank all the subjects for participating in this study and all the researchers and collaborators who participated in this study.

## Conflict of interest

The authors declare that the research was conducted in the absence of any commercial or financial relationships that could be construed as a potential conflict of interest.

## Publisher’s note

All claims expressed in this article are solely those of the authors and do not necessarily represent those of their affiliated organizations, or those of the publisher, the editors and the reviewers. Any product that may be evaluated in this article, or claim that may be made by its manufacturer, is not guaranteed or endorsed by the publisher.

## References

[1] Sun H Saeedi P Karuranga S Pinkepank M Ogurtsova K Duncan BB. IDF diabetes atlas: Global, regional and country-level diabetes prevalence estimates for 2021 and projections for 2045. Diabetes Res Clin Pract (2022) 183:109119. doi: 10.1016/j.diabres.2021.109119 34879977 PMC11057359

[2] Chatterjee S Khunti K Davies MJ. Type 2 diabetes. Lancet (2017) 389(10085):2239–51. doi: 10.1016/S0140-6736(17)30058-2 28190580

[3] Goni MH Markussis V Tolis G. Octreotide effect on ovarian morphology in insulin-resistant PCOS patients following six-month decapeptyl treatment. Am J Reprod Immunol (1994) 31(2-3):104–11. doi: 10.1111/j.1600-0897.1994.tb00854.x 8049019

[4] Desai D Mehta D Mathias P Menon G Schubart UK. Health care utilization and burden of diabetic ketoacidosis in the U.S. over the past decade: A nationwide analysis. Diabetes Care (2018) 41(8):1631–8. doi: 10.2337/dc17-1379 29773640

[5] Galindo RJ Zambrano C Vellanki P. Comment on desai et al. health care utilization and burden of diabetic ketoacidosis in the U.S. over the past decade: A nationwide analysis. Diabetes Care (2018) 41:1631–8. doi: 10.2337/dc17-1379 29773640

[6] Chen J Zeng H Ouyang X Zhu M Huang Q Yu W. The incidence, risk factors, and long-term outcomes of acute kidney injury in hospitalized diabetic ketoacidosis patients. BMC Nephrol (2020) 21(1):48. doi: 10.1186/s12882-020-1709-z 32050921 PMC7017527

[7] Benoit SR Hora I Pasquel FJ Gregg EW Albright AL Imperatore G. Trends in emergency department visits and inpatient admissions for hyperglycemic crises in adults with diabetes in the U.S., 2006-2015. Diabetes Care (2020) 43(5):1057–64. doi: 10.2337/dc19-2449 PMC7171947 32161050

[8] Dhatariya KK Glaser NS Codner E Umpierrez GE. Diabetic ketoacidosis. Nat Rev Dis Primers (2020) 6(1):40. doi: 10.1038/s41572-020-0165-1 32409703

[9] Saidi P Gurney CW. Attenuation of urinary erythropoietin activity under various conditions. J Lab Clin Med (1970) 76(4):659–67. doi: 10.5555/uri:pii:0022214370902520 5458028

[10] Wlodarek D. Role of ketogenic diets in neurodegenerative diseases (Alzheimer's disease and parkinson's disease). Nutrients (2019) 11(1):169. doi: 10.3390/nu11010169 PMC6356942 30650523

[11] Rusek M Pluta R Ulamek-Koziol M Czuczwar SJ. Ketogenic diet in alzheimer's disease. Int J Mol Sci (2019) 20(16):3892. doi: 10.3390/ijms20163892 PMC6720297 31405021

[12] Kanikarla-Marie P Jain SK. Hyperketonemia and ketosis increase the risk of complications in type 1 diabetes. Free Radic Biol Med (2016) 95:268–77. doi: 10.1016/j.freeradbiomed.2016.03.020 PMC4867238 27036365

[13] Kitabchi AE Umpierrez GE Miles JM Fisher JN. Hyperglycemic crises in adult patients with diabetes. Diabetes Care (2009) 32(7):1335–43. doi: 10.2337/dc09-9032 PMC2699725 19564476

[14] Collins GS Reitsma JB Altman DG Moons KG. Transparent reporting of a multivariable prediction model for individual prognosis or diagnosis (TRIPOD): The TRIPOD statement. Bmj (2015) 350:g7594. doi: 10.1161/CIRCULATIONAHA.114.014508 25569120

[15] Jiang S Fang J Yu T Liu L Zou G Gao H. Novel model predicts diabetic nephropathy in type 2 diabetes. Am J Nephrol (2020) 51(2):130–8. doi: 10.1159/000505145 31865340

[16] Yu D Shang J Cai Y Wang Z Zhang X Zhao B. Derivation and external validation of a risk prediction algorithm to estimate future risk of cardiovascular death among patients with type 2 diabetes and incident diabetic nephropathy: prospective cohort study. BMJ Open Diabetes Res Care (2019) 7(1):e000735. doi: 10.1136/bmjdrc-2019-000735 PMC6861120 31798896

[17] Ehrmann D Kulzer B Roos T Haak T Al-Khatib M Hermanns N. Risk factors and prevention strategies for diabetic ketoacidosis in people with established type 1 diabetes. Lancet Diabetes Endocrinol (2020) 8(5):436–46. doi: 10.1016/S2213-8587(20)30042-5 32333879

[18] Vellanki P Umpierrez GE. Diabetic ketoacidosis: A common debut of diabetes among african americans with type 2 diabetes. Endocr Pract (2017) 23(8):971–8. doi: 10.4158/EP161679.RA PMC6092188 28534682

[19] Ren X Mou W Su C Chen X Zhang H Cao B. Increase in peripheral blood intermediate monocytes is associated with the development of recent-onset type 1 diabetes mellitus in children. Int J Biol Sci (2017) 13(2):209–18. doi: 10.7150/ijbs.15659 PMC5332875 28255273

[20] Marathe CS Rayner CK Jones KL Horowitz M. Relationships between gastric emptying, postprandial glycemia, and incretin hormones. Diabetes Care (2013) 36(5):1396–405. doi: 10.2337/dc12-1609 PMC3631884 23613599

[21] Hallberg SJ McKenzie AL Williams PT Bhanpuri NH Peters AL Campbell WW. Effectiveness and safety of a novel care model for the management of type 2 diabetes at 1 year: An open-label, non-randomized, controlled study. Diabetes Ther (2018) 9(2):583–612. doi: 10.1007/s13300-018-0373-9 29417495 PMC6104272

[22] Su JB Zhao LH Zhang XL Cai HL Huang HY Xu F. HbA1c variability and diabetic peripheral neuropathy in type 2 diabetic patients. Cardiovasc Diabetol (2018) 17(1):47. doi: 10.1186/s12933-018-0693-0 29598819 PMC5874999

[23] Spiller S Blüher M Hoffmann R. Plasma levels of free fatty acids correlate with type 2 diabetes mellitus. Diabetes Obes Metab (2018) 20(11):2661–9. doi: 10.1111/dom.13449 29943387

[24] Boden G Shulman GI. Free fatty acids in obesity and type 2 diabetes: defining their role in the development of insulin resistance and beta-cell dysfunction. Eur J Clin Invest (2002) 32(Suppl 3):14–23. doi: 10.1046/j.1365-2362.32.s3.3.x 12028371

[25] Zhang M Li Y Cui W Yang P Li H Sheng C. THE CLINICAL AND METABOLIC CHARACTERISTICS OF YOUNG-ONSET KETOSIS-PRONE TYPE 2 DIABETES IN CHINA. Endocr Pract (2015) 21(12):1364–71. doi: 10.4158/EP15778.OR 26372299

[26] Moller N Foss AC Gravholt CH Mortensen UM Poulsen SH Mogensen CE. Myocardial injury with biomarker elevation in diabetic ketoacidosis. J Diabetes Complications (2005) 19(6):361–3. doi: 10.1016/j.jdiacomp.2005.04.003 16260354

[27] Gao L Li Y Fei D Ma L Chen S Feng B. Prevalence of and risk factors for diabetic ketosis in Chinese diabetic patients with random blood glucose levels >13.9 mmol/L: Results from the CHina study in prEvalence of diabetiC ketosis (CHECK) study. J Diabetes (2018) 10(3):249–55. doi: 10.1111/1753-0407 28685968

[28] Wang J Zhang M Liu Z Wang X Pang Y Lu Y. Heterogeneous clinical features of ketosis-prone type 2 diabetes mellitus patients: Gender, age, loss of weight and HbA1c. Minerva Endocrinol (2019) 44(4):351–6. doi: 10.23736/S0391-1977.18.02925-5 30482009

[29] Magliano DJ Sacre JW Harding JL Gregg EW Zimmet PZ Shaw JE. Young-onset type 2 diabetes mellitus - implications for morbidity and mortality. Nat Rev Endocrinol (2020) 16(6):321–31. doi: 10.1038/s41574-020-0334-z 32203408

[30] Nanayakkara N Curtis AJ Heritier S Nanayakkara N Curtis AJ Heritier S Gadowski AM Pavkov ME Kenealy T. Impact of age at type 2 diabetes mellitus diagnosis on mortality and vascular complications: systematic review and meta-analyses. Diabetologia (2021) 64(2):275–87. doi: 10.1007/s00125-020-05319-w PMC7801294 33313987

[31] Zhu B Bu L Zhang M Gusdon AM Zheng L Rampersad S. HbA(1c) as a screening tool for ketosis in patients with type 2 diabetes mellitus. Sci Rep (2016) 6:39687. doi: 10.1038/srep39687 28009017 PMC5180185

[32] Yuan X Wang J Yang S Gao M Cao L Li X. Effect of the ketogenic diet on glycemic control, insulin resistance, and lipid metabolism in patients with T2DM: A systematic review and meta-analysis. Nutr Diabetes (2020) 10(1):38. doi: 10.1038/s41387-020-00142-z 33257645 PMC7705738

[33] Rao P Wang H Fang H Gao Q Zhang J Song M. Association between IGF2BP2 polymorphisms and type 2 diabetes mellitus: A case-control study and meta-analysis. Int J Environ Res Public Health (2016) 13(6):574. doi: 10.3390/ijerph13060574 PMC4924031 27294943

